# Supplementary material for: Dosing Evaluation of Ceftazidime–Avibactam in Intensive Care Unit Patients Based on Pharmacokinetic/Pharmacodynamic (PK/PD) Modeling and Simulation
Source: Antibiotics (Basel). 2024 Sep 9;13(9):861. doi: 10.3390/antibiotics13090861 (PMC11429409; doi:10.3390/antibiotics13090861)
Supplement: Supplementary file 1 [file antibiotics-13-00861-s001.zip › antibiotics-3160560-supplementary.pdf]

antibiotics

Supplementary Material

Dosing evaluation of ceftazidime/avibactam in intensive care unit patients based on pharmacokinetic-pharmacodynamic (PK/PD) modelling and simulation.

Hinojal Zazo [1,2], Yuridia Aguazul [1] and José M. Lanao [1,2*]

[1] Area of Pharmacy and Pharmaceutical Technology, Pharmaceutical Sciences Department, University of Salamanca, Spain.; HZG: hinojal@usal.es and JML: jmlanao@usal.es
[2] Institute of Biomedical Research of Salamanca (IBSAL), Spain
* Correspondence: jmlanao@usal.es; Tel: +34 923 294 518

Supplementary Material

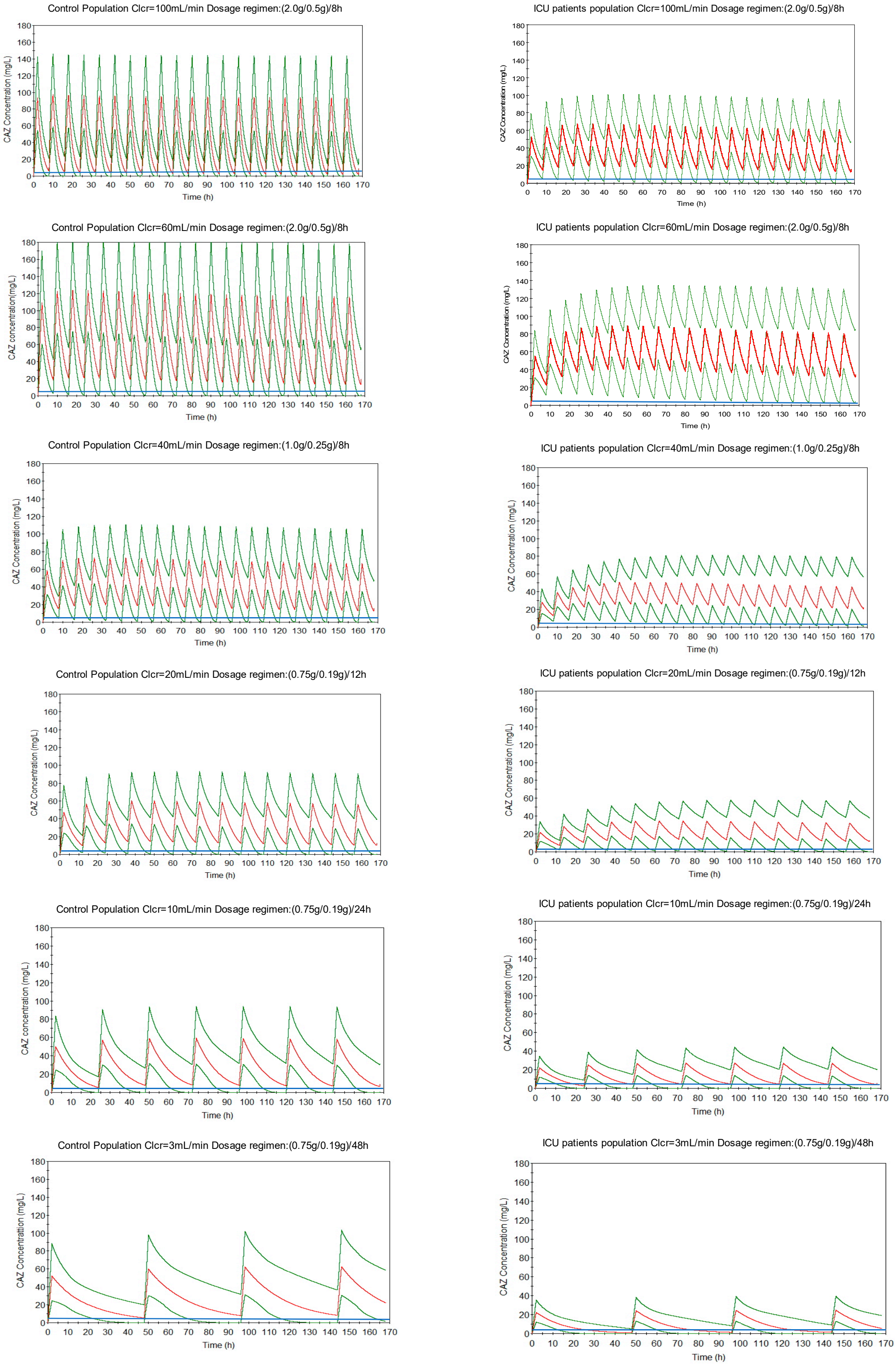

Figure S1: Ceftazidime (CAZ) concentrations simulated along the week of treatment for the dosage regimens included in the SmPC for control (A) and ICU (B) populations with different degree of renal function (1: Clcr of 100 mL/min; 2: Clcr of 60 mL/min; 3: Clcr of 40 mL/min; 4: Clcr of 20 mL/min; 5: Clcr of 10 mL/min; 6: Clcr of 3 mL/min;); Blue line MIC: 4 mg/L

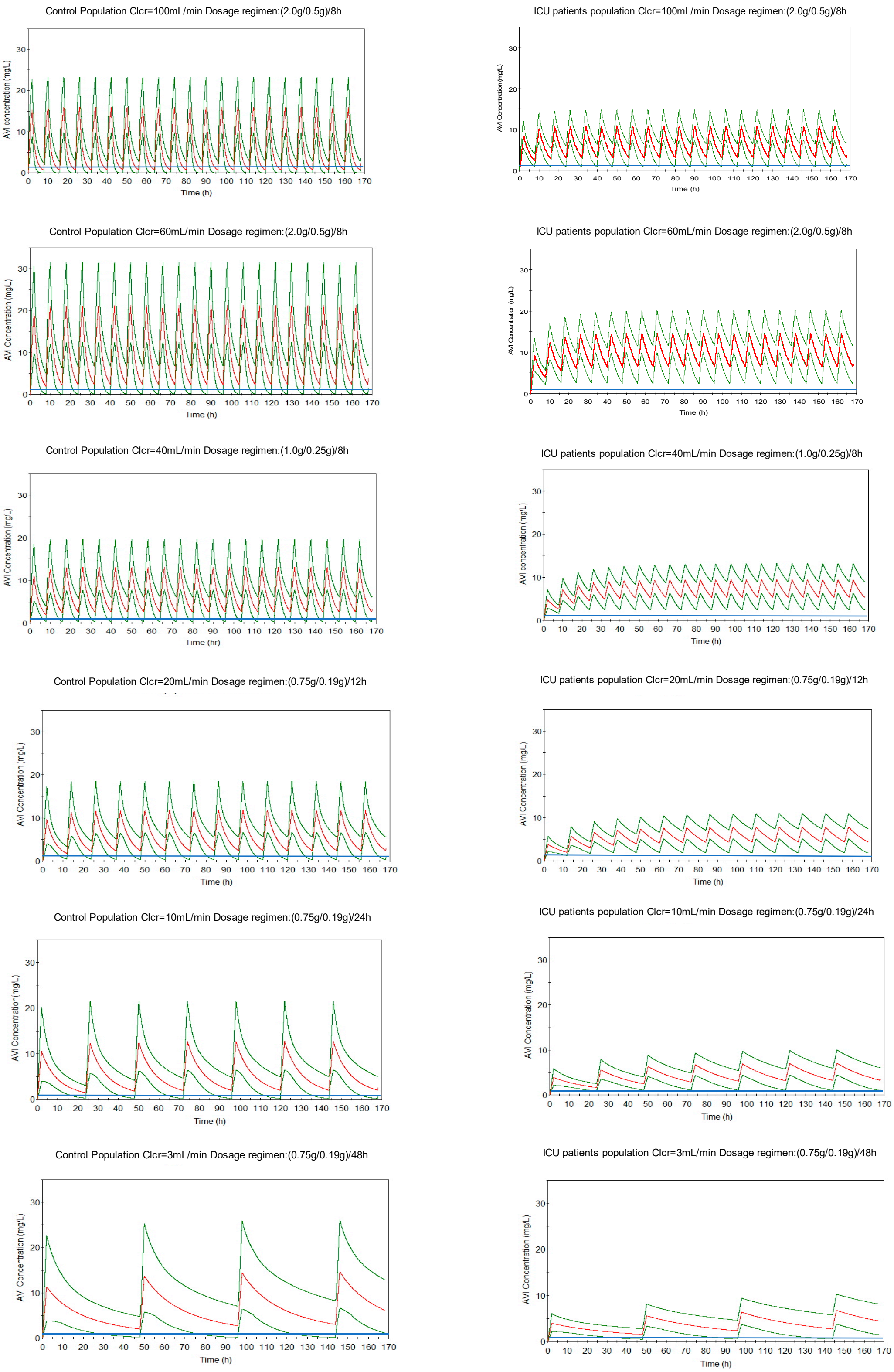

Figure S2: Avibactam (AVI) concentrations simulated along the week of treatment for the dosage regimens included in the SmPC for control (A) and ICU (B) populations with different degree of renal function (1: Clcr of 100 mL/min; 2: Clcr of 60 mL/min; 3: Clcr of 40 mL/min; 4: Clcr of 20 mL/min; 5: Clcr of 10 mL/min; 6: Clcr of 3 mL/min;); Blue line: MIC (1 mg/L)

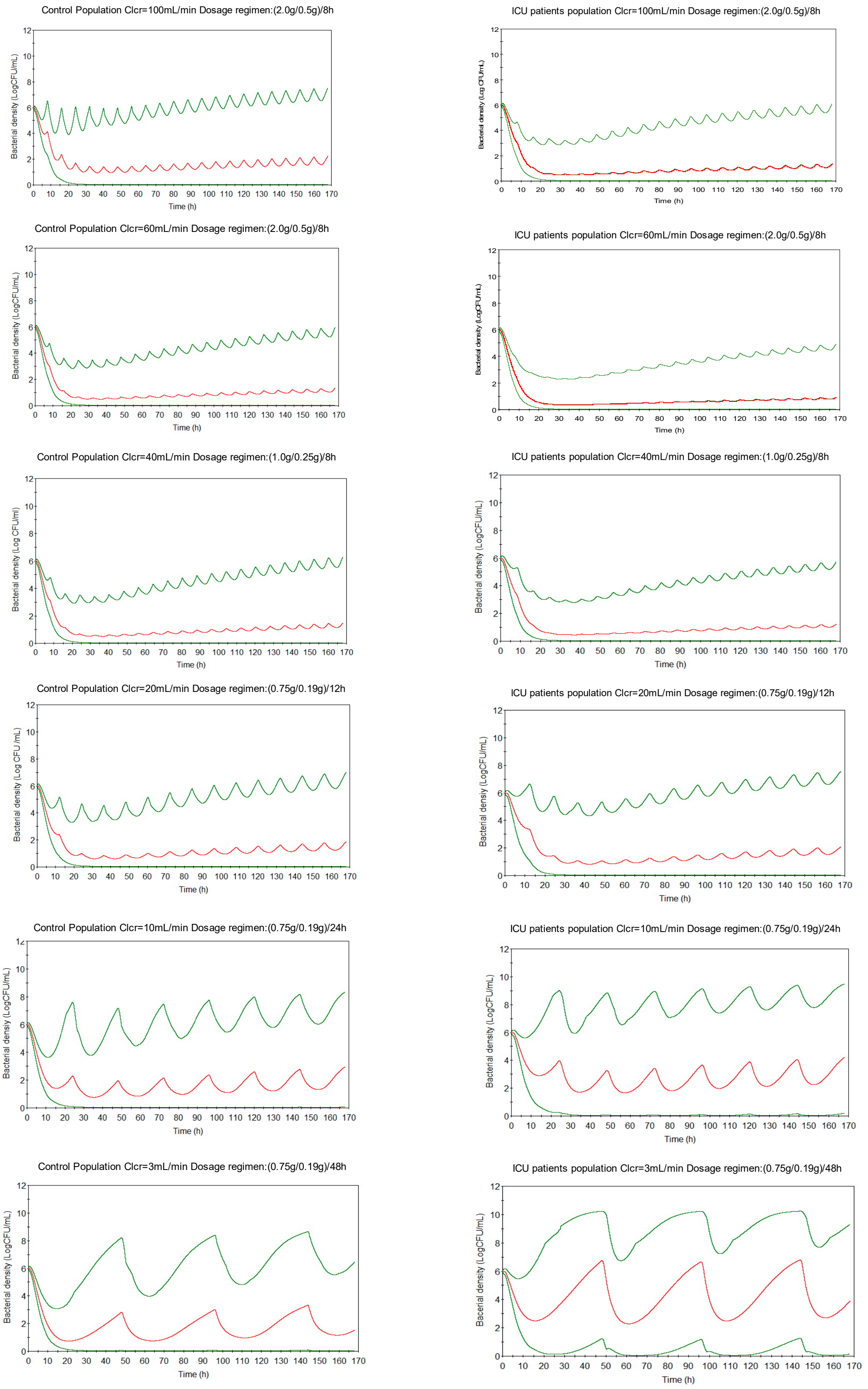

Figure S3 Bacterial density simulated along the week of treatment for the dosage regimens included in the SmPC for control (A) and ICU (B) populations with different degree of renal function (1: Clcr of 100 mL/min; 2: Clcr of 60 mL/min; 3: Clcr of 40 mL/min; 4: Clcr of 20 mL/min; 5: Clcr of 10 mL/min; 6: Clcr of 3 mL/min;);

Table S1. Simulated Ceftazidime and Avibactam maximum and minimum concentrations at steady-state for the dosage regimens included in the SmPC and suggested.

Dosage regimen	Population	Clcr (mL/min)	Dosage Regimen ((CAZ g/AVI g)/h)	Ceftazidima C_{max}^{ss} (mg/L) [CV (%)]	Ceftazidima C_{min}^{ss} (mg/L) [CV (%)]	Avibactam C_{max}^{ss} (mg/L) [CV (%)]	Avibactam C_{min}^{ss} (mg/L) [CV (%)]
Summary of Product Characteristics	Control	100	(2.0/0.5)/8	95.9 [27.1]	3.2 [232]	15.9 [25.8]	0.70 [134]
		60	(2.0/0.5)/8	123 [27.6]	17.7 [117]	21.2 [27.3]	2.36 [91.1]
		40	(1.0/0.25)/8	73.3 [28.2]	17.0 [98.8]	13.1 [27.6]	2.61 [69.3]
		20	(0.75/0.19)/12	59.6 [31.4]	13.6 [102]	11.8 [31.0]	2.40 [66.2]
		10	(0.75/0.19)/24	59.5 [32.4]	8.16 [126]	12.6 [37.2]	1.87 [74.3]
		3	(0.75/0.19)/48	62.6 [35.6]	8.3 [153]	14.7 [41.2]	2.98 [86.6]
	ICU	100	(2.0/0.5)/8	65.4 [28.6]	17.8 [90.0]	10.9 [20.7]	3.19 [53.9]
		60	(2.0/0.5)/8	87.1 [33.2]	39.0 [63.3]	14.6 [21.1]	6.57 [42.3]
		40	(1.0/0.25)/8	50.2 [33.9]	25.7 [64.2]	9.41 [21.9]	5.30 [36.8]
		20	(0.75/0.19)/12	34.6 [35.5]	14.0 [81.4]	7.61 [21.9]	4.20 [35.9]
		10	(0.75/0.19)/24	27.3 [33.0]	4.71 [130]	6.70 [22.8]	2.85 [40.3]
		3	(0.75/0.19)/48	24.8 [33.0]	1.41 [257]	6.76 [29.4]	2.73 [59.7]
Suggested	Control	10	(0.75/0.19)/12	87.6 [31.9]	40.4 [62.2]	16.5 [29.6]	6.52 [48.7]
		3	(0.75/0.19)/24	89.9 [40.1]	34.5 [92.9]	20.3 [39.1]	7.60 [58.1]
	ICU	10	(0.75/0.19)/12	44.8 [36.2]	24.0 [63.4]	10.7 [24.7]	7.21 [32.7]
		3	(0.75/0.19)/24	33.3 [41.5]	10.5 [118]	10.7 [33.9]	6.86 [49.1]

CLcr: creatinine clearance; CV: coefficient of variation; ICU: Intensive care unit.

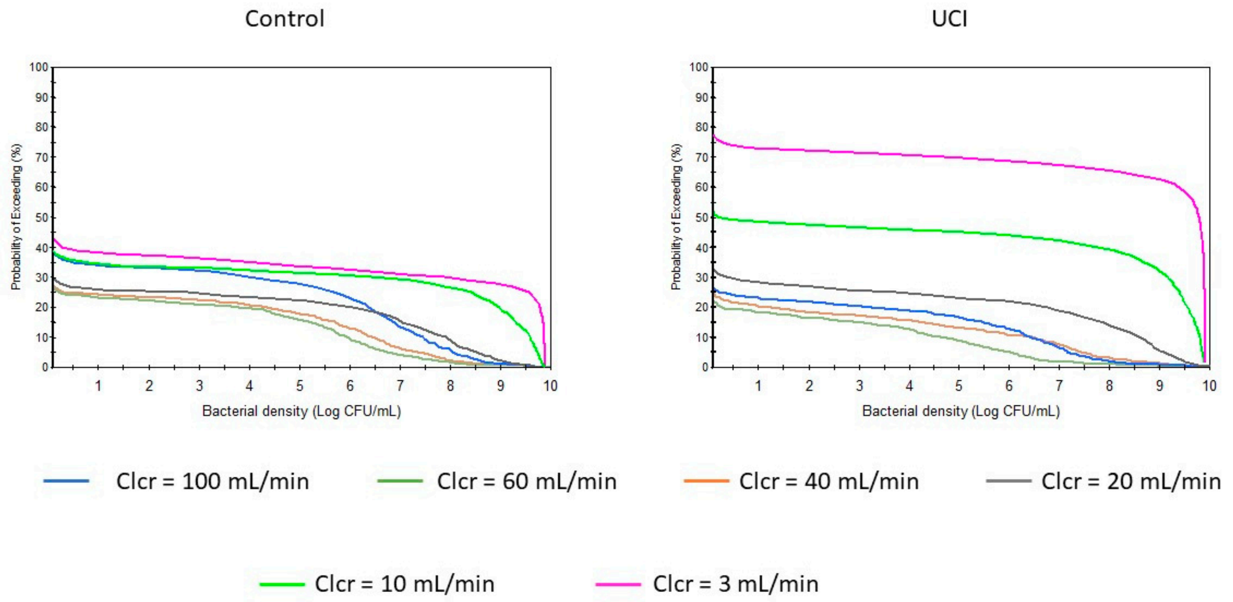

Figure S4. Probability of exceeding a bacterial density value with different simulated dosage regimens for control and ICU patients.

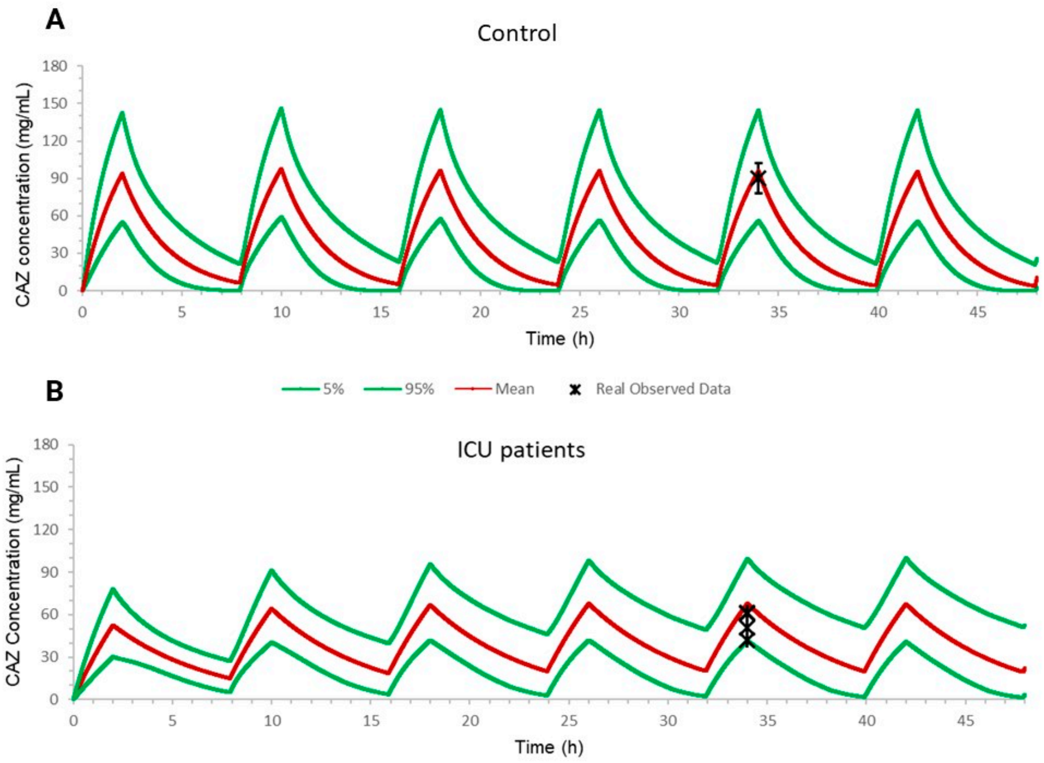

Figure S5. Visual Predicted Check for control and ICU patients. Lines: Simulated concentrations (green lines: 5/95 IP and red line: mean), Black cross: Observed data from Falcone M. et al, Fresan D. et al and Sy SKB et al. [7,11,12]
